# Albendazole Monotherapy and Spleen Preservation in an Adolescent with Isolated Primary Splenic Cystic Echinococcosis: A Case Report

**DOI:** 10.3390/pathogens15080846

**Published:** 2026-08-14

**Authors:** Andrea Marino, Alessandro Franzò, Concetta Ippolito, Giovanni Cacciaguerra, Giuseppe Nunnari

**Affiliations:** 1 Infectious Diseases Unit, Department of Clinical and Experimental Medicine, ARNAS Garibaldi Hospital, University of Catania, 91122 Catania, Italy; alefranzosr@gmail.com (A.F.); giuseppe.nunnari1@unict.it (G.N.); 2 Radiology Unit, ARNAS Garibaldi Hospital, 91122 Catania, Italy; cippolito@arnasgaribaldi.it; 3 Section of Pediatrics and Child Neuropsychiatry, Department of Child and Experimental Medicine, University of Catania, 95100 Catania, Italy; gio.cacciaguerra@gmail.com

**Keywords:** cystic echinococcosis, hydatid cyst, spleen, albendazole, spleen-preserving management, children, WHO-IWGE classification

## Abstract

Cystic echinococcosis (CE), caused by *Echinococcus granulosus* sensu lato, most commonly involves the liver and lungs, whereas the spleen is an uncommon site, accounting for less than 2% of cases; isolated primary splenic involvement is rarer still and is only sporadically reported in children. Total splenectomy has traditionally been the standard treatment for splenic CE, yet it carries a lifelong risk of overwhelming post-splenectomy infection, a particularly relevant concern in a child. We describe a 13-year-old boy from a rural, sheep-rearing area of eastern Sicily in whom a splenic cyst was incidentally detected on ultrasonography performed after minor blunt abdominal trauma. Ultrasound and contrast-enhanced computed tomography showed a 3.7 cm cyst with a partly calcified wall and a thin enhancing rim compatible with a pericyst, reported as stage CE3a (WHO-IWGE), and *Echinococcus* IgG serology was positive. In the absence of histological confirmation, a presumptive diagnosis of splenic CE was made after considering the relevant differential diagnoses. Given the small, uncomplicated cyst and the wish to preserve splenic function, the patient was managed with continuous albendazole monotherapy (400 mg twice daily) for six months, without any invasive procedure. Treatment was well tolerated, and serial imaging showed a modest reduction in size together with evolution of the cyst content toward a solid, calcified, inactive-appearing pattern that remained stable for six months after treatment discontinuation. This case suggests that, in exceptionally selected patients, albendazole monotherapy with close imaging surveillance may allow spleen preservation; it should not be regarded as a standard alternative to surgery.

## 1. Introduction

Cystic echinococcosis (CE) is a neglected zoonotic infection caused by the larval stage of *Echinococcus granulosus* sensu lato, and remains endemic across the Mediterranean basin, including several regions of Italy such as Sicily and Sardinia, where traditional sheep farming sustains the parasite life cycle [1,2,3]. Humans are accidental intermediate hosts, and cysts may develop slowly over years, frequently remaining asymptomatic until they are discovered incidentally or produce mass-related symptoms [1,2,3].

The liver (approximately 70% of cases) and the lungs (around 20%) are by far the most common locations. The spleen is the third most frequently affected organ but accounts for less than 2% of cases, and primary isolated splenic hydatid disease—without concomitant hepatic or other organ involvement—is distinctly uncommon [4,5,6]. Splenic CE is even more rarely reported in children and adolescents, in whom published pediatric CE series are dominated by hepatic and pulmonary disease [7].

The diagnosis of CE is essentially image-based. Ultrasonography is the reference modality and underpins the WHO Informal Working Group on Echinococcosis (WHO-IWGE) standardized classification, which stratifies cysts into active (CE1–CE2), transitional (CE3a–CE3b), and inactive (CE4–CE5) stages; computed tomography adds anatomical detail and is particularly useful for extrahepatic and calcified lesions [1,3,8]. Serology is a useful adjunct but has variable sensitivity that is lower for extrahepatic and inactive cysts, and it cannot reliably confirm parasitological cure [1,9].

Management follows a stage-specific, organ-oriented strategy comprising surgery, percutaneous techniques (e.g., PAIR), anti-infective drug therapy with albendazole, and a “watch-and-wait” approach for inactive cysts [1,3]. For the spleen, however, the evidence base is limited and largely surgical, and no validated treatment algorithm exists [3,10]. Building on this organ-preserving rationale, and by analogy with the non-operative management of selected asymptomatic hepatic cysts [11], we report the exclusively medical, spleen-preserving management of an isolated primary splenic CE cyst in an adolescent.

## 2. Case Presentation

A 13-year-old boy was referred to our infectious diseases outpatient clinic in the spring of 2023 for evaluation of an incidentally detected splenic cystic lesion. The lesion had been identified on an abdominal ultrasound performed in the pediatric emergency setting after minor blunt abdominal trauma, to exclude traumatic splenic injury. He was asymptomatic and afebrile, without left upper-quadrant pain. He lived in a rural area of eastern Sicily and had regular direct contact with sheep, an epidemiological setting consistent with CE transmission. His past medical history was unremarkable; he took no regular medications and reported no allergies.

On examination he was in good general condition (weight 71 kg, height 164 cm, body mass index 26.4 kg/m^2^), with normal vital signs. A grade 2/6 mitral systolic murmur with occasional extrasystoles prompted cardiologic evaluation; echocardiography was normal, excluding cardiac involvement. The abdomen was soft and non-tender, without palpable organomegaly or lymphadenopathy.

Laboratory testing showed a near-normal blood count with relative lymphocytosis and no eosinophilia, mild hyperbilirubinemia (total bilirubin 1.5 mg/dL), and normal transaminases, pancreatic enzymes, renal function, and glucose. *Echinococcus* IgG serology was positive (index 3.05; positive if >1).

**Figure 1:**
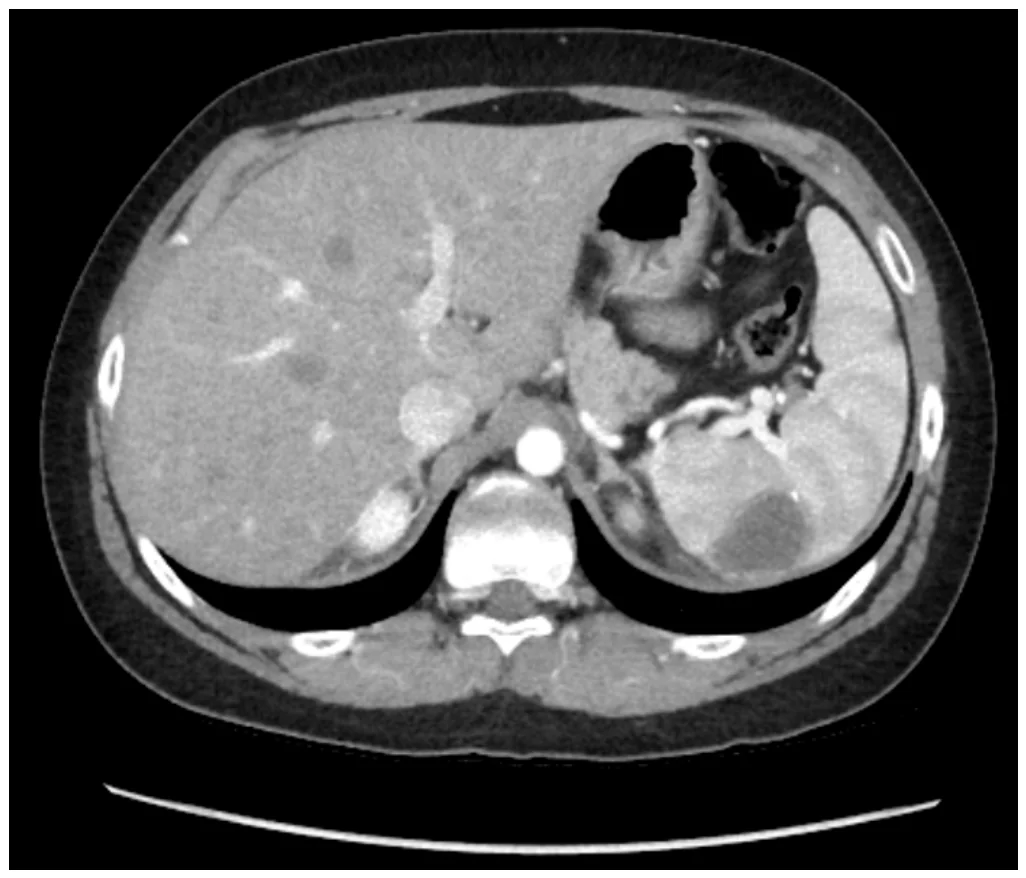
Contrast-enhanced computed tomography of the abdomen showing, at the upper pole of the spleen, an ovalar cystic lesion of fluid density (~3.7 × 2.5 cm) with thin peripheral calcifications and a regular enhancing pericystic rim, compatible with a splenic hydatid cyst.

Abdominal ultrasound demonstrated a normal-sized spleen containing a well-defined rounded lesion with lobulated margins and coarse peripheral calcifications, composed of anechoic lacunae alternating with an isoechoic component, measuring up to 3.7 × 2.7 cm; it was reported by the radiologist as stage CE3a (transitional) according to the WHO-IWGE classification. No detached endocyst membrane (“water-lily” sign) was identified; the CE3a designation therefore reflected the radiologist’s interpretation of a mixed-echogenicity, partly calcified cyst rather than a pathognomonic finding. Contrast-enhanced computed tomography of the chest and abdomen confirmed, at the upper pole of the spleen, an ovalar lesion of 3.7 × 2.5 cm of fluid density surrounded by thin peripheral calcifications, with a regular ~4 mm hypodense enhancing rim compatible with a pericyst; the appearances were considered consistent with a splenic hydatid cyst. There was no hepatic, pulmonary, or other organ involvement and no lymphadenopathy (Figure 1).

In the absence of histological or parasitological confirmation—percutaneous sampling being deliberately avoided because of the risks of cyst-content spillage and anaphylaxis—a presumptive diagnosis of isolated primary splenic CE was made. A post-traumatic pseudocyst was considered unlikely because the coarse mural calcification indicates a chronic lesion pre-dating the recent minor trauma and because pseudocysts lack a true (parasitic) wall, an enhancing pericyst, and positive serology; a congenital/epidermoid cyst is typically anechoic with a thin, non-calcified wall and negative serology; a lymphangioma is usually multiseptate without a calcified wall; an organized hematoma would be expected to evolve or resolve on serial imaging; and a splenic abscess was excluded by the absence of fever, leukocytosis, or raised inflammatory markers. The differential diagnosis of a splenic cystic lesion includes post-traumatic pseudocyst (of particular relevance here, as the lesion was found during trauma work-up), congenital/epidermoid cyst, lymphangioma, organized hematoma, and abscess; CE was favored on the basis of the positive *Echinococcus* serology in a patient with direct ovine exposure in an endemic area, the partly calcified cyst wall, the enhancing pericystic rim on CT, and the subsequent morphological evolution under albendazole. After multidisciplinary discussion, and given the small (<5 cm), uncomplicated, asymptomatic cyst and the explicit goal of preserving splenic function in a child, exclusively medical management with albendazole was chosen in preference to splenectomy, spleen-preserving surgery, or PAIR. PAIR was set aside because its efficacy and safety are established mainly for hepatic CE and are not defined for splenic cysts, and because splenic puncture carries risks of bleeding and of cyst-content spillage; a small cyst amenable to drug therapy did not warrant an invasive procedure [1,3,12]. Because the continuous schedule differs from the cyclical regimen in the product label, treatment was administered off-label, with written informed consent obtained from the patient’s parent.

The patient received albendazole 400 mg twice daily with food continuously from 16 April to 16 October 2023 (six months), under structured monitoring with complete blood count, aminotransferases, gamma-glutamyl transferase, bilirubin, creatinine, and C-reactive protein at approximately monthly intervals. Therapy was well tolerated, with no hepatotoxicity, no cytopenia, and no other adverse effects; liver enzymes and renal function remained within normal limits throughout (Table 1). A continuous rather than cyclical schedule was used, consistent with current evidence and expert opinion favouring uninterrupted administration [13]. At 71 kg the dose (800 mg/day) corresponded to approximately 11 mg/kg/day, within the 10–15 mg/kg/day range recommended for children and used in pediatric albendazole cohorts and monotherapy series, with a common ceiling of 800 mg/day [14,15].

**Figure 2:**
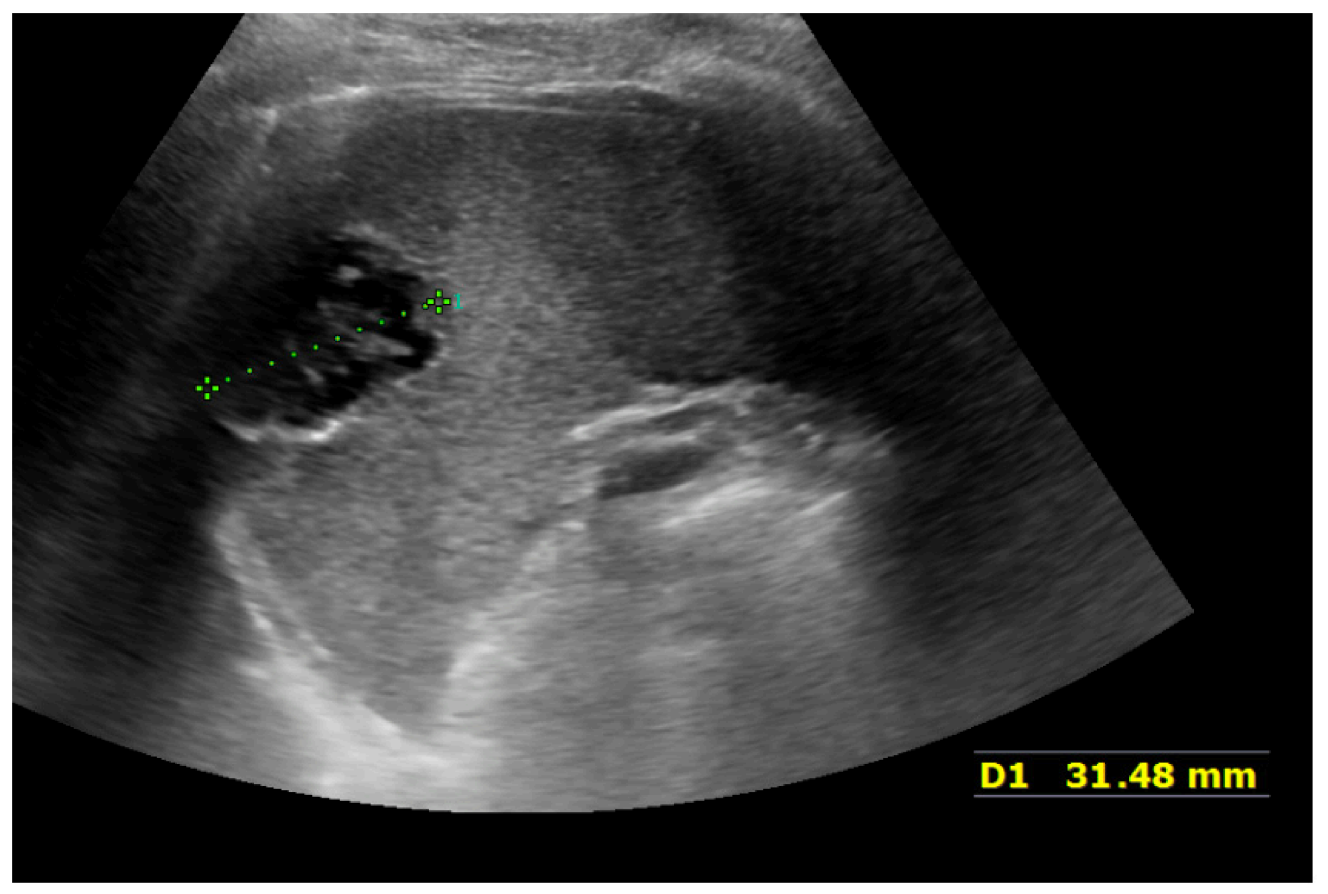
Follow-up grayscale (B-mode) splenic ultrasound (April 2024, six months after treatment). Within a normal-sized spleen with homogeneous parenchyma, a well-defined rounded lesion (calipers) is seen with a partly calcified wall and heterogeneous, predominantly solid hyperechoic content and coarse internal echogenic foci consistent with calcification, without an anechoic fluid component or detached endocyst membranes. The appearances are those of an inactive-appearing hydatid cyst (WHO-IWGE CE4–CE5).

Serial imaging documented a favorable evolution. The maximal cyst diameter decreased from 3.7 × 2.7 cm at baseline to 2.9 × 2.4 cm at three months, and the cyst content changed progressively: the walls became thicker and partly hyperechoic, and by the end of treatment the lesion appeared solid with calcified walls and hyperechoic internal material—features consistent with a transition from an active/transitional cyst toward an inactive-appearing stage. Although the change in diameter was modest and may be influenced by scan plane and operator variability, the morphological evolution was the more informative finding and was concordant across examinations. Importantly, this inactive-appearing pattern remained stable on ultrasound performed six months after albendazole discontinuation (3 × 2.6 cm; April 2024) (Figure 2), over a total imaging follow-up of approximately 13 months. Throughout this period the patient remained asymptomatic, the spleen was preserved and normal in size, and there was no reactivation, enlargement, or complication. The most recent evaluation available for this report was in April 2024. Clinical and ultrasonographic surveillance is ongoing.

## 3. Discussion

This case is notable on three counts: the rarity of the site, the pediatric setting, and the exclusively medical, spleen-preserving strategy. Primary isolated splenic CE accounts for less than 2% of all cases of hydatid disease, and although the spleen is the third most commonly affected organ, isolated splenic involvement without hepatic or other localization is uncommon and only sporadically reported in children and adolescents [4,5,6,7]. Endemic Mediterranean areas, including Sicily, continue to report pediatric CE, underscoring the need to keep hydatid disease in the differential diagnosis of a splenic cystic lesion even in younger patients [1,2,7,16]. As summarized in Table 2, the published splenic CE literature is dominated by surgical management—total splenectomy or spleen-preserving surgery—with albendazole used almost exclusively as a peri-operative adjunct; in a dedicated splenic series no patient received medication alone, and pediatric albendazole-monotherapy series have not included splenic cysts [10,12,14,15,17]. A closely comparable adolescent—also with a history of minor abdominal trauma—required albendazole plus total splenectomy because the cyst was giant [18]. Italian splenic cases continue to be reported from Sicily and Sardinia [19]. Exclusively medical, spleen-preserving management of a splenic cyst, as in our patient, therefore appears very rarely reported and constitutes the principal contribution of this report.

Our diagnosis rested on characteristic imaging combined with positive serology rather than on histological confirmation, and the differential diagnosis of a splenic cystic lesion—including post-traumatic pseudocyst, congenital/epidermoid cyst, lymphangioma, organized hematoma, and abscess—was explicitly considered before attributing the lesion to CE. Ultrasonography, complemented by contrast-enhanced computed tomography, is the cornerstone of diagnosis: a calcified wall and an enhancing pericystic rim are supportive features, and CT is particularly useful for extrahepatic and calcified cysts [1,3,8]. Serology provided supportive evidence, although its sensitivity is variable and generally lower for extrahepatic and inactive cysts, and a positive titer cannot by itself confirm activity, viability, or subsequent cure [1,9]. For the same reason, serology was not used as a follow-up tool: anti-*Echinococcus* IgG may persist for years after successful treatment and correlates poorly with cyst inactivation, so imaging—not serology—is the recommended monitoring modality [1,3,9]. Two caveats deserve emphasis and temper diagnostic and staging certainty: the WHO-IWGE stage-specific algorithm was validated for hepatic CE and does not necessarily apply to the spleen, and wall calcification per se does not indicate inactivity, so the reported CE3a stage is best regarded as a radiological interpretation rather than an established fact [3]. We therefore consider the diagnosis presumptive, albeit well supported by the epidemiological context, imaging, serology, and treatment response.

The management decision reflected a deliberate choice to preserve splenic function. In hepatic CE, small active or transitional cysts smaller than 5 cm may be treated with albendazole monotherapy, larger cysts with percutaneous techniques under drug cover, and inactive cysts with a watch-and-wait approach [1,3,11]. Splenic CE has traditionally been treated by total splenectomy, and some series continue to favor it, particularly in resource-limited settings [10]; however, the lifelong susceptibility to overwhelming post-splenectomy infection by encapsulated bacteria—especially consequential in a child—has driven growing interest in spleen-preserving surgery such as partial splenectomy, cystectomy, and deroofing [11,20]. In a small, asymptomatic cyst, we extended this organ-preserving philosophy to fully non-operative management with albendazole alone, conceptually analogous to the non-operative management of selected asymptomatic hepatic cysts [11] and consistent with the broader shift toward less invasive, function-preserving treatment of pediatric CE [21].

Albendazole is typically administered continuously for three to six months or longer, with the best responses in small, active cysts, and requires monitoring for hepatotoxicity and marrow suppression [1,3,13]. A continuous regimen, rather than the older cyclical courses, is now supported by evidence and expert opinion [13]. The weight-adjusted dose used here (~11 mg/kg/day; 800 mg/day) was concordant with the 10–15 mg/kg/day recommended in pediatric practice and with doses reported in pediatric cohorts and monotherapy series [14,15]. Reports of albendazole-induced hepatotoxicity necessitating interruption or substitution—including in isolated splenic CE—underline the importance of laboratory surveillance; our patient completed six months of continuous therapy without biochemical or haematological toxicity. The imaging response—size reduction with progressive solidification and calcification of the cyst content, sustained after treatment—corresponds to the expected evolution toward an inactive stage rather than to complete disappearance, and reinforces that treatment response in CE is judged primarily by serial imaging, not by serology [1,3].

This report has several limitations. It describes a single patient; the diagnosis was presumptive and not confirmed histologically, albeit for sound clinical reasons; the WHO-IWGE stage assignment is uncertain at an extrahepatic site; only two orthogonal cyst diameters were available from routine clinical scans, without volumetric measurement; and the imaging response consisted of inactivation and stabilization rather than resolution. The follow-up, although it now includes six months of off-treatment stability, remains limited, and because CE can reactivate, continued clinical and ultrasonographic surveillance is essential. Within these constraints, the case adds to the sparse evidence supporting exclusively medical, spleen-preserving management of small, uncomplicated splenic CE cysts, particularly in children, and argues for individualized, multidisciplinary, organ-preserving decision-making. Prospective data and multicenter registries are needed to define which splenic cysts can be managed safely without surgery.

## 4. Conclusions

Isolated primary splenic cystic echinococcosis is rare, particularly in children. In a carefully selected adolescent with a small, uncomplicated splenic cyst and a high level of diagnostic confidence, albendazole monotherapy with close imaging surveillance allowed the spleen to be preserved, was well tolerated, and was followed by imaging evidence of cyst inactivation that remained stable after treatment discontinuation. This experience suggests that exclusively medical management may be considered in exceptionally selected patients when multidisciplinary follow-up is available; it should not be interpreted as establishing a standard alternative to surgery, and continued surveillance and larger studies remain necessary.

## Figures and Tables

**Table 1:** Clinical, laboratory, and imaging timeline. US, ultrasound; CT, computed tomography; WBC, white blood cells; N, neutrophils; L, lymphocytes; AST, aspartate aminotransferase; ALT, alanine aminotransferase; GGT, gamma-glutamyl transferase; CRP, C-reactive protein; eGFR, estimated glomerular filtration rate; BID, twice daily; WHO-IWGE, WHO Informal Working Group on Echinococcosis.

Date	Clinical Event/Visit	Key Laboratory Data	Imaging/Cyst Status
March 2023	Incidental finding after minor blunt abdominal trauma; asymptomatic	—	US: splenic lesion 3.7 × 2.7 cm, lobulated margins, coarse peripheral calcifications, anechoic lacunae + isoechoic component; reported as CE3a (WHO-IWGE)
April 2023	Diagnostic work-up; normal echocardiography	WBC 6.2 × 10^9^/L (N 33%, L 52%); no eosinophilia; total bilirubin 1.5 mg/dL; normal AST/ALT, pancreatic enzymes, creatinine. *Echinococcus* IgG positive (index 3.05; positive > 1)	Contrast-enhanced CT: upper-pole splenic ovalar lesion 3.7 × 2.5 cm, fluid density, thin peripheral calcifications; regular ~4 mm enhancing rim compatible with a pericyst. No hepatic/pulmonary/other involvement.
16 April 2023	Albendazole 400 mg BID with food started; continuous (off-label) schedule; written parental consent	—	—
April–May 2023	On therapy, well tolerated	AST 24–29, ALT 37–38, GGT 14–23, total bilirubin 1.2–1.3 mg/dL, creatinine 0.42–0.46 mg/dL; normal CBC	—
July 2023	On therapy (3 months)	Normal CBC and liver/renal panel	US: lesion 2.9 × 2.4 cm (reduced), thicker partly hyperechoic walls; spleen normal (10 cm)
16 October 2023	Albendazole completed (6 months total)	Normal CBC, transaminases, renal function, CRP	—
October 2023	Post-treatment assessment	Hb 13.2 g/dL, WBC 4.7 × 10^9^/L, normal transaminases and CRP	US: lesion ~3 cm, calcific walls, hyperechoic internal content (inactive-appearing)
April 2024	Follow-up (6 months off therapy); asymptomatic	Normal CBC and liver panel; creatinine 0.76 mg/dL (eGFR 136 mL/min)	US: lesion 3 × 2.6 cm, unchanged; spleen normal. Ongoing clinical + US surveillance

**Table 2:** Selected reports of splenic cystic echinococcosis and of albendazole treatment in children, and the role of medical therapy. n.r., not reported; n/a, not applicable (no splenic localization/no splenectomy performed); M, male.

Study (Year)	Design; Age/Sex	Cyst (Size; Stage)	Treatment	Spleen Preserved	Follow-Up; Outcome
Present case (2026)	Case report; 13 y, M	3.7 cm; CE3a → CE4–CE5	Albendazole monotherapy, 6 months	Yes	~13 mo; inactivation, stable
Vatankhah et al. (2024) [18]	Case report; 16 y, M (minor trauma)	Giant; n.r.	Albendazole + total splenectomy	No	Uneventful recovery
Mejri et al. (2021) [10]	Case series (n = 8); adults	Isolated splenic	Total splenectomy (4)/spleen-preserving surgery (4) + peri-operative albendazole	Partial	Favoured splenectomy
Korkut et al. (2022) [12]	Retrospective series; adults	Splenic CE	Surgery (spleen-preserving preferred) + peri-operative albendazole; none drug-only	Mostly	No recurrence
Aljaiuossi et al. (2024) [17]	Cohort; adults	Primary splenic	Minimally invasive spleen-preserving surgery + albendazole	Yes	Good; low recurrence
Shmueli et al. (2023) [15]	Case series (n = 18 children)	Liver/lung/kidney; no splenic	Albendazole monotherapy	n/a	72% size reduction; 22% resolution
Moroni et al. (2016) [14]	Pediatric cohort	Abdominal CE; no splenic-specific	Albendazole	n/a	Variable radiological response

## Data Availability

The original contributions presented in this study are included in the article/Supplementary Material. Further inquiries can be directed to the corresponding author.

## Supplementary Materials

The following supporting information can be downloaded at: https://www.mdpi.com/article/10.3390/pathogens15080846/s1. Supplementary Table S1: CARE checklist.
