# Supplementary material for: Albendazole Monotherapy and Spleen Preservation in an Adolescent with Isolated Primary Splenic Cystic Echinococcosis: A Case Report
Source: Pathogens. 2026 Aug 14;15(8):846. doi: 10.3390/pathogens15080846 (PMC13516974; doi:10.3390/pathogens15080846)
Supplement: Supplementary file 1 [file pathogens-15-00846-s001.zip › pathogens-4500818-supplementary.pdf]

CARE Checklist

Information included in: "Albendazole Monotherapy and Spleen Preservation in an Adolescent with Isolated Primary Splenic Cystic Echinococcosis: A Case Report."

Topic	Item	Checklist item description	Reported in (section / anchor)
**Title**	1	The diagnosis or intervention of primary focus followed by the words "case report"	Title — "Albendazole Monotherapy and Spleen Preservation … Isolated Primary Splenic Cystic Echinococcosis: A Case Report"
**Key Words**	2	2 to 5 key words identifying diagnoses or interventions, including "case report"	Keywords — cystic echinococcosis; hydatid cyst; spleen; albendazole; spleen-preserving management; children; WHO-IWGE classification. [See note a]
**Abstract**	3a	Introduction: what is unique about this case and what does it add to the literature?	Abstract — closing sentences (exclusively medical, spleen-preserving management; contribution over surgery)
**(no references)**	3b	Main symptoms and/or important clinical findings	Abstract — incidental splenic cyst after minor blunt trauma; asymptomatic child
	3c	The main diagnoses, therapeutic interventions, and outcomes	Abstract — presumptive splenic CE (CE3a); continuous albendazole 6 months; size reduction and inactivation, stable off-treatment
	3d	Conclusion — the main "take-away" lesson(s)	Abstract — final sentence (monotherapy may allow spleen preservation in selected patients; not a standard alternative to surgery)
**Introduction**	4	One or two paragraphs on why this case is unique (may include references)	Introduction — final paragraph (organ-preserving rationale; exclusively medical management of isolated splenic CE)
**Patient Information**	5a	De-identified patient-specific information	Case Presentation — ¶1 (13-year-old boy, rural eastern Sicily)
	5b	Primary concerns and symptoms of the patient	Case Presentation — ¶1 (incidentally detected cyst on trauma work-up; asymptomatic, afebrile)
	5c	Medical, family, and psycho-social history including relevant genetic information	Case Presentation — ¶1 (unremarkable PMH; no medications/allergies; regular sheep contact). Genetic information not applicable
	5d	Relevant past interventions with outcomes	Case Presentation — ¶1 (none; no prior interventions or regular medications)
**Clinical Findings**	6	Significant physical examination and important clinical findings	Case Presentation — ¶2 (general condition, BMI; grade 2/6 murmur with normal echocardiography; soft, non-tender abdomen)
**Timeline**	7	Historical and current information organized as a timeline	Table 1 (clinical, laboratory, and imaging timeline, Mar 2023 – Apr 2024)
**Diagnostic Assessment**	8a	Diagnostic testing (PE, laboratory, imaging, surveys)	Case Presentation — ¶3–4 (blood count, biochemistry, Echinococcus IgG; ultrasound; contrast-enhanced CT); Figures 1–2; Table 1
	8b	Diagnostic challenges (access, financial, cultural, etc.)	Case Presentation — diagnosis paragraph (no histological/parasitological confirmation; percutaneous sampling deliberately avoided). Discussion — diagnosis paragraph (variable serology; WHO-IWGE staging not validated at extrahepatic sites)
	8c	Diagnosis (including other diagnoses considered)	Case Presentation — diagnosis paragraph (differential: post-traumatic pseudocyst, congenital/epidermoid cyst, lymphangioma, organized hematoma, abscess; basis for favoring CE)

Topic	Item	Checklist item description	Reported in (section / anchor)
	**8d**	Prognosis (such as staging) where applicable	Case Presentation / Table 1 / Figure 2 (WHO-IWGE CE3a → inactive-appearing CE4–CE5)
**Therapeutic Intervention**	**9a**	Types of therapeutic intervention (pharmacologic, surgical, etc.)	Case Presentation — treatment paragraph (pharmacologic: albendazole monotherapy; no invasive procedure)
	**9b**	Administration of therapeutic intervention (dosage, strength, duration)	Case Presentation — treatment paragraph (albendazole 400 mg twice daily with food, continuous, 16 Apr – 16 Oct 2023, six months; ≈11 mg/kg/day)
	**9c**	Changes in therapeutic intervention (with rationale)	Not applicable — no dose changes; continuous (vs cyclical) schedule completed as planned (rationale in treatment paragraph and Discussion)
**Follow-up and Outcomes**	**10a**	Clinician- and patient-assessed outcomes (if available)	Case Presentation — follow-up paragraph (asymptomatic; spleen preserved and normal-sized; no complication). Discussion
	**10b**	Important follow-up diagnostic and other test results	Case Presentation — follow-up paragraph; Table 1 (serial ultrasound; stable inactive-appearing lesion, April 2024)
	**10c**	Intervention adherence and tolerability (how assessed)	Case Presentation — treatment paragraph (approximately monthly CBC, aminotransferases, GGT, bilirubin, creatinine, CRP; well tolerated)
	**10d**	Adverse and unanticipated events	Case Presentation — treatment paragraph (none; no hepatotoxicity, cytopenia, or other adverse effects)
**Discussion**	**11a**	Scientific discussion of strengths AND limitations	Discussion — limitations paragraph (single case; presumptive diagnosis; staging uncertainty; two orthogonal diameters only; inactivation rather than resolution)
	**11b**	Discussion of the relevant medical literature with references	Discussion — throughout; Table 2 (comparison with published splenic CE and pediatric albendazole reports)
	**11c**	Scientific rationale for any conclusions (assessment of possible causes)	Discussion — diagnosis and management paragraphs (imaging-based inactivation; organ-preserving rationale)
	**11d**	Primary “take-away” lessons (without references), one-paragraph conclusion	Conclusions section
**Patient Perspective**	**12**	The patient shares their perspective on the treatment(s) received	Not reported — optional CARE item; no patient/parent perspective paragraph included. [See note b]
**Informed Consent**	**13**	Did the patient give informed consent? Provide if requested	YES — written informed consent for publication obtained from the patient’s parent (minor); stated in the Informed Consent Statement and the case narrative
